# Gut Microbiota and Psychiatric Disorders: A Two-Sample Mendelian Randomization Study

**DOI:** 10.3389/fmicb.2021.737197

**Published:** 2022-02-04

**Authors:** Jing-Jing Ni, Qian Xu, Shan-Shan Yan, Bai-Xue Han, Hong Zhang, Xin-Tong Wei, Gui-Juan Feng, Min Zhao, Yu-Fang Pei, Lei Zhang

**Affiliations:** ^1^Jiangsu Key Laboratory of Preventive and Translational Medicine for Geriatric Diseases, Medical College of Soochow University, Suzhou, China; ^2^Center for Genetic Epidemiology and Genomics, School of Public Health, Medical College of Soochow University, Suzhou, China; ^3^Department of Epidemiology and Biostatistics, School of Public Health, Medical College of Soochow University, Suzhou, China

**Keywords:** Mendelian randomization (MR), gut microbiota (GM), psychiatric disorders, causal relationship, species *Clostridium innocuum*

## Abstract

Evidence supports the observational associations of gut microbiota with a variety of psychiatric disorders, but the causal nature of such associations remains obscure. Aiming to comprehensively investigate their causal relationship and to identify specific causal microbe taxa for psychiatric diseases, we conducted a two-sample Mendelian randomization (MR) analysis of gut microbiome with 15 psychiatric diseases. Specifically, the microbiome genome-wide association study (GWAS) in 18,473 individuals from the MiBioGen study was used as exposure sample, and the GWAS for 15 psychiatric diseases was used as outcome samples. One-hundred ninety bacterial taxa from six levels were available for analysis. At a multiple-testing corrected significance level (phylum *P* < 5.56 × 10^–3^, class *P* < 3.33 × 10^–3^, order *P* < 2.63 × 10^–3^, family *P* < 1.67 × 10^–3^, genus *P* < 4.90 × 10^–4^, and species *P* < 3.33 × 10^–3^), the following eight causal associations from seven bacterial features (one phylum + three classes + one order + one family + one species) were identified: family *Prevotellaceae* with autism spectrum disorder (*P* = 5.31 × 10^–4^), class *Betaproteobacteria* with bipolar disorder (*P* = 1.53 × 10^–3^), class *Actinobacteria* with schizophrenia (*P* = 1.33 × 10^–3^), class *Bacteroidia* and order *Bacteroidales* with Tourette syndrome (*P* = 2.51 × 10^–3^ and 2.51 × 10^–3^), phylum *Actinobacteria* and class *Actinobacteria* with extroversion (*P* = 8.22 × 10^–4^ and 1.09 × 10^–3^), and species *Clostridium innocuum* with neuroticism (*P* = 8.92 × 10^–4^). Sensitivity analysis showed no evidence of reverse causality, pleiotropy, and heterogeneity. Our findings offered novel insights into the gut microbiota–mediated development mechanism of psychiatric disorders.

## Introduction

Psychiatric disorders are a cluster of complex psychological syndromes in cognition, behavior, or emotion regulation, representing the second leading cause of disability and premature death worldwide (Whiteford et al., 2013). Epidemiological research has shown that the global lifetime incidence of psychiatric disorders in adults ranges between 12.2 and 48.6%, with the overall prevalence varying from 4.3 to 26.4% (Demyttenaere et al., 2004). Certain mental diseases, such as depression, attention deficit/hyperactivity disorder (ADHD), autism spectrum disorder (ASD), and schizophrenia (SCZ), account for approximately 12% of the global disease burden (Ochoa-Reparaz et al., 2020). Because they frequently require long-term treatment, their burden was estimated to be $8.5 trillion in 2010 and continuously raised by 41% between 1990 and 2010 (Patel et al., 2016). Thus, there is an urgent need to identify potential causal risk factors for various psychiatric disorders.

The etiologies of psychiatric disorders are largely multi-factorial, including psychological, genetic, and environmental factors. Recently, growing evidence has suggested that the gut microbiota is closely related to host health and is involved in the etiology of a variety of human complex diseases including psychiatric disorders (Chow et al., 2010; Clemente et al., 2012; Cryan et al., 2019). The gut microbiota is a dynamic and complex community of ecological microbes, inhabiting the human intestine, even called a “forgotten organ” (O’Hara and Shanahan, 2006). The microbiota and central nervous system might communicate with each other *via* the microbiota–gut–brain (MGB) axis, which includes diverse routes including the immune response, the vagus and enteric nerve, and microbiota-derived molecules or metabolites (Cryan et al., 2019). A variety of observational studies have shown that the gut microbiome differs between healthy controls and psychiatric patients (Wang et al., 2011; Strati et al., 2017; Valles-Colomer et al., 2019; Hua et al., 2020). Altered compositions and function of intestinal microbiota were observed in ASD or depression patients (Strati et al., 2017; Valles-Colomer et al., 2019). Further experimental studies also demonstrated the importance of microbiota in the development of psychiatric disorders. For example, fecal microbiota transplantation (FMT) from human donors with ASD into murine induced exacerbated corresponding symptoms (Sharon et al., 2019). However, the causal association between the gut microbiota and psychiatric disorders remains unclear.

Conventionally, the gold standard for inferring a causal association is randomized controlled trials (RCTs). Whereas a RCT is difficult to implement or sometimes even impossible due to ethic restriction. As an alternative, Mendelian randomization (MR) is an efficient method to statistically assess causality from an exposure to an outcome, utilizing genetic variants as instrumental variables (IVs) (Katan, 1986; Smith and Ebrahim, 2003). Because a random assortment of genetic variants occurs during meiosis yielding according to the Mendel’s second law, the selected genetic variants avoid social economic confounding (Emdin et al., 2017). The MR approach is conceptually similar to the RCT study, with only one difference that patients are allocated according to their DNA genotypes. MR analysis relies on three important assumptions: (i) IV is strongly associated with exposure; (ii) IV should be independent of any observed and unobserved confounders of exposure–outcome association; (iii) IV–outcome association is only mediated *via* exposure rather than any other pathway. In a recent study, utilizing MR, Sanna et al. (2019) identified that propionate, one type of fecal short-chain fatty acid (SCFA), increases the risk of type 2 diabetes, demonstrating the efficacy of microbiota-oriented causal inference *via* MR analysis.

Two-sample MR analysis can utilize single-nucleotide polymorphism (SNP)–exposure and SNP–outcome associations from independent GWAS analyses and combine them into a single causal estimate. As the number of genome-wide association studies (GWASs) in gut microbiota and psychiatric disorders has increased rapidly, large-scale summary statistics have become more widely available (Demontis et al., 2019; Grove et al., 2019; Howard et al., 2019; Stahl et al., 2019; Kurilshikov et al., 2021), allowing for two-sample MR analysis with significantly improved statistical power.

In this study, we applied a systematic two-sample MR analysis to comprehensively explore whether gut microbiota components have a causal effect on various psychiatric disorders and to identify specific causal bacterial taxa. Specifically, summary statistics of gut microbiota and 15 common psychiatric disorders/traits were derived from large-scale GWAS or genetic consortia.

## Materials and Methods

### Data Sources

**FIGURE 1:**
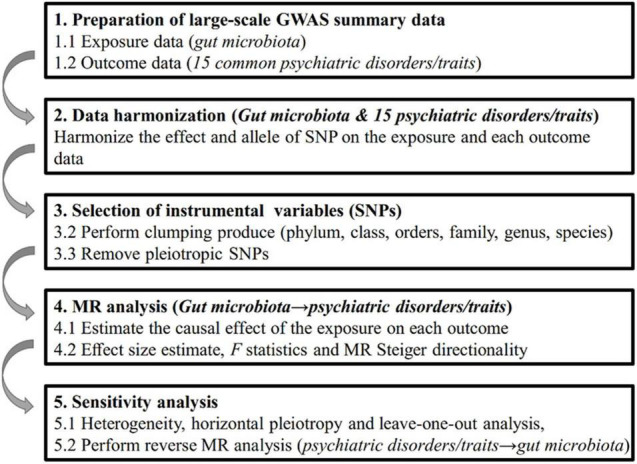
Diagrammatic description of the whole workflow in MR analysis. A flowchart of the whole MR analysis was displayed in this figure.

Genome-wide association study summary-level statistics for gut microbiota and 15 common psychiatric disorders/traits were obtained from previous studies or consortia. All studies were approved by their respective institutional review boards (IRBs). No new IRB approval was required. A flowchart briefly presents the whole procedure in Figure 1.

Genome-wide association study summary statistics of gut microbiota was assessed from the MiBioGen study (Kurilshikov et al., 2021) (^1^, as of June 28, 2020), which is the largest, multi-ethnic, genome-wide meta-analysis of the gut microbiome to date. Briefly, the MiBioGen study coordinated 16S rRNA gene sequencing profiles and whole-genome genotyping data from 18,473 individuals (25 cohorts) as described elsewhere (Kurilshikov et al., 2021). The microbial composition of distinct cohorts was profiled by targeting three different variable regions of the 16S rRNA gene: V4, V3–V4, and V1–V2, and all microbiome datasets were rarefied to 10,000 reads per cohort. The majority of cohorts used similar imputation procedures using the Michigan Imputation Server or IMPUTE2 software and the Haplotype Reference Consortium 1.0 or 1.1 reference panel. Then, microbiome trait loci mapping was performed to identify genetic loci that affect the relative abundance of microbial taxa. The cutoffs mapping included at least 3,000 effective samples in the presence of at least three cohorts. In total, all available GWAS summary statistics of 190 bacterial taxa were eventually included in the MR analysis.

Genome-wide association study summary statistics for psychiatric disorders were generated from large-scale GWAS or their meta-analysis. We collected as many psychiatric disorders as possible, resulting in 15 disorders (Schizophrenia Working Group of the Psychiatric Genomics Consortium, 2014; van den Berg et al., 2016; International Obsessive Compulsive Disorder Foundation Genetics Collaborative (Iocdf-Gc) and Ocd Collaborative Genetics Association Studies (OCGAS), 2018; Nagel et al., 2018; Pasman et al., 2018; Demontis et al., 2019; Grove et al., 2019; Howard et al., 2019; Meier et al., 2019; Nievergelt et al., 2019; Sanchez-Roige et al., 2019; Stahl et al., 2019; Watson et al., 2019; Yu et al., 2019; Erlangsen et al., 2020). Criteria to define these disorders are listed in Supplementary Table 1. For each disorder, summary statistics from the largest GWAS were assessed. Detailed descriptions of GWAS for 15 common psychiatric disorders/traits, including the ethnicity, genotyping platform, imputation reference panel, and consortium, are presented in Table 1.

**TABLE 1:** Characteristics of included genome-wide association studies for psychiatric disorders.

Psychiatric disorders/traits	Ethnicity	N	No. SNP	Data type	Genotyping platform and SNP panel	References	Study
Depression	European	500,199	8,483,301	Binary	Affymetrix United Kingdom Biobank/BiLEVE Axiom array, IMPUTE4, HRC and UK10K; PGC Ricopili (Schizophrenia Working Group of the Psychiatric Genomics Consortium, 2014)	Howard et al. (2019)	UKB, PGC
ADHD	Multi-ancestry	55,374	8,047,421	Binary	Illumina PsychChip; 1KGP Phase 3; PGC Ricopili	Demontis et al. (2019)	iPSYCH, PGC
ASD	European	46,351	9,112,386	Binary	PsychChip array; 1KGP Phase 3; PGC Ricopili	Grove et al. (2019)	iPSYCH, PGC
BD	European	51,710	9,372,253	Binary	PGC Ricopili, 1KGP	Stahl et al. (2019)	PGC
SCZ	Multi-ancestry	152,805	9,444,230	Binary	PGC Ricopili, 1KGP	Schizophrenia Working Group of the Psychiatric Genomics Consortium (2014)	PGC
AUDIT	European	121,604	16,213,998	Continuous	Affymetrix United Kingdom Biobank/BiLEVE Axiom array, IMPUTE4, HRC	Sanchez-Roige et al. (2019)	UKB
CUD	European	162,082	11,535,592	Continuous	Affymetrix United Kingdom Biobank/BiLEVE Axiom array, IMPUTE4, HRC; various, 1KGP Phase 1	Pasman et al. (2018)	UKB, ICC
AN	European	72,517	82,191,012	Binary	Affymetrix United Kingdom Biobank/BiLEVE Axiom array, IMPUTE4, HRC; PGC Ricopili, 1KGP Phase 3; various	Watson et al. (2019)	UKB, PGC, ANGI, GCAN/WTCCC3
TS	European	14,307	8,265,318	Binary	Illumina HumanOmniExpress 8/12v1, IMPUTE v2, 1KGP Phase 1	Yu et al. (2019)	PGC
OCD	Multi-ancestry	9,725	8,409,516	Binary	Illumina Human610-Quadv1_B, Illumina HumanOmniExpress, IMPUTE2, 1KGP Phase 1	International Obsessive Compulsive Disorder Foundation Genetics Collaborative (Iocdf-Gc) and Ocd Collaborative Genetics Association Studies (OCGAS) (2018)	IOCDF-GC, OCGAS
Extroversion	European	63,030	6,941,603	Continuous	Illumina/Affymetrix, IMPUTE, 1KGP Phase 1	van den Berg et al. (2016)	GPC
NEU	European	390,278	10,849,319	Continuous	Affymetrix United Kingdom Biobank/BiLEVE Axiom array, HRC and UK10K; Illumina/Affymetrix, IMPUTE, 1KGP Phase 1	Nagel et al. (2018)	UKB, GPC
AD	European	23,809	9,029,716	Binary	Illumina PsychChip, SHAPEIT and IMPUTE2, PGC Ricopili, 1KGP phase 3	Meier et al. (2019)	iPSYCH
SD	European	29,056	8,047,611	Binary	Illumina PsychChip, SHAPEIT and IMPUTE2, PGC Ricopili, 1KGP phase 3	Meier et al. (2019)	iPSYCH
PTSD	Multi-ancestry	206,655	9,788,621	Binary	Affymetrix Axiom array; Illumina genotyping arrays, PGC Ricopili, IMPUTE2, 1KGP phase 3	Nievergelt et al. (2019)	UKB, PGC
SA	European	50,264	8,017,027	Binary	Infinium PsychChip v1.0 array, IMPUTE2, 1KGP phase 3	Erlangsen et al. (2020)	iPSYCH

*No. SNP is the total number of SNPs released from the summary data of GWAS.*

*“Various” refers to more details on genotyping platform as described elsewhere previously.*

*ADHD, attention deficit/hyperactivity disorder; ASD, autism spectrum disorder; BD, bipolar disorder; SCZ, schizophrenia; AUDIT, alcohol use disorder identification test; CUD, cannabis use disorder; AN, anorexia nervosa; TS, Tourette syndrome; OCD, obsessive-compulsive disorder; NEU, neuroticism; AD, anxiety-related disorder; SD, stress-related disorder; PTSD, posttraumatic stress disorder; SA, suicide attempts; HRC, Haplotype Reference Consortium; PGC, Psychiatric Genomics Consortium; Ricopili, Rapid Imputation Consortium Pipeline; UKB, United Kingdom Biobank; 1KGP, 1000 Genomes Project; iPSYCH, Integrative Psychiatric Research; ICC, International Cannabis Consortium; ANGI, Anorexia Nervosa Genetics Initiative GCAN/WTCCC3, Genetic Consortium for Anorexia Nervosa/Wellcome Trust Case Control Consortium-3; IOCDF-GC, International Obsessive Compulsive Disorder Foundation Genetics Collaborative; OCGAS, Collaborative Genetics Association Studies; GPC: Genetics of Personality Consortium.*

### Instrumental Variable Selection

Bacterial taxa were analyzed at six levels (phylum, class, order, family, genus, and species). A distinct taxon was defined as a feature. Candidate IVs for each feature were selected at the *P* < 1.0 × 10^–5^ significance in accordance with the study of Sanna et al. (2019). Then, SNPs associated with each feature were clumped with PLINK (v1.9) to retain only independent SNPs. The linkage disequilibrium (LD) threshold was set to be *r*^2^< 0.1, with a clumping window of 500 kb. The 1,000 Genomes Project sequencing data (phase 3) was used to estimate LD.

The horizontal pleiotropy effect, that is, the confounding effect caused by other diseases, is a severe problem and may violate the second assumption in MR analysis. We applied the MR-PRESSO test and the MR-Egger regression test to monitor potential horizontal pleiotropy effect. The MR-PRESSO Outlier test calculates for each SNP a *P*-value for its pleiotropy significance, whereas the MR-PRESSO Global test calculates a *P*-value for overall horizontal pleiotropy. SNPs were sorted in an ascending order in terms of their MR-PRESSO Outlier test *P*-values and were then removed one by one. Each time a SNP was removed from the list, the MR-PRESSO Global test was performed on the remaining SNPs. The recursion was repeated until *P*-value for the Global test was unsignificant (*P* > 0.05). The list of the remaining SNPs after removing pleiotropic ones was used for subsequent MR analysis. The significant intercept item of MR-Egger implies the existence of pleiotropy.

To avoid distortion of strand orientation or allele coding, we deleted palindromic SNPs (e.g., with A/T or G/C alleles). In the harmonization process, we aligned alleles to the human genome reference sequence (build 37) and removed ambiguous and duplicated SNPs.

### Effect Size Estimate

The GWAS summary statistics of gut microbiota and psychiatric disorders/traits were derived from a standardized phenotype (i.e., mean 0 and variance 1). Therefore, we could estimate the proportion of phenotypic variance explained by SNP from summary statistics with the formula 2*f*(1−*f*)β^2^, where *f* is the effect allele frequency and β is the regression coefficient for gut microbiota and psychiatric disorders/traits.

### Mendelian Randomization Analysis

We performed MR analysis to investigate the causal relationship between microbiome features and the 15 common psychiatric disorders/traits. For features containing only one IV, the Wald ratio test was used to estimate the association between the identified IV and each psychiatric disorder/trait (Burgess et al., 2017). For features containing multiple IVs, five popular MR methods were used: the inverse-variance weighted (IVW) test (Burgess et al., 2013), the maximum likelihood estimator (MLE) (Pierce and Burgess, 2013), the MR-Egger regression (Bowden et al., 2015), the weighted median estimator (WME) (Bowden et al., 2016), and the MR-PRESSO (Verbanck et al., 2018). Each statistical method has its own model assumption, and any violation of the assumption may make the method inferior or even completely invalid. Specific to the five methods investigated: 1) The IVW method assumes no horizontal pleiotropy (Burgess et al., 2013); 2) the MLE (Pierce and Burgess, 2013) assumes the linear correlation of outcome and exposure with jointly normal distribution and allows for uncertainty in both gene–exposure and gene–outcome associations (Burgess et al., 2015); 3) the MR-Egger assumes the presence of pleiotropy in > 50% SNPs (Bowden et al., 2015); 4) the WME assumes the presence of pleiotropy in < 50% SNPs (Bowden et al., 2016); and 5) the MR-PRESSO assumes the presence of pleiotropy but will remove pleiotropic SNPs intrinsically (Verbanck et al., 2018). The IVW method is reported to be slightly more powerful than the others under certain conditions (Bowden et al., 2016). Therefore, the results were mainly based on the IVW method, with the other four methods serving as its complements. Additionally, we established a multiple-testing significance threshold at each feature level (phylum, class, order, family, genus, and species) defined as *P* < 0.05/*n* (where *n* is the effective number of independent bacterial taxa on the corresponding taxonomic level).

To assess robustness of significant results, we performed several sensitivity analyses. The potential heterogeneity was examined by the Q test in the IVW test and the MR-Egger regression. Meanwhile, the leave-one-out analysis was performed to determine whether the causal signal was driven by one SNP. To infer causal direction, we used the MR Steiger directionality (Hemani et al., 2017) test to examine whether the exposure was directionally causal for the outcome. This approach compares the variance explained by IVs for both exposure and outcome. If the IVs explain a greater variance in the exposure than the outcome, then the identified causal association could be considered directionally credible. Furthermore, we calculated *F* statistics (Burgess and Thompson, 2011) to evaluate the weak instrument bias using the following formula:


$$F=\frac{R^{2}\,\left(n-1-k\right)}{\left(1-R^{2}\right)\,k},$$

where *n*, *k*, and *R*^2^ are sample size, number of IVs, and the variance explained by IVs, respectively. An *F*-value less than 10 indicates weak instrument.

### Bidirectional Mendelian Randomization Analysis

We performed an additional reverse MR analysis to explore reverse causality. Significant reverse MR analysis indicates reverse causality from psychiatric disorders/traits (as exposure) to microbiota features (as outcome). The reverse MR analysis procedure was the same as the above MR analysis.

All of the analyses, including MR analyses and sensitivity analyses, were performed with the R packages *TwoSampleMR*^2^ (Hemani et al., 2018) and *MRPRESSO*^3^ (Verbanck et al., 2018).

## Results

After removing palindromic SNPs, we identified 937, 1,576, 1,583, 2,390, 6,525, and 739 SNPs associated with gut microbiota in the phylum, class, order, family, genus, and species levels at the suggestive significance level *P* < 1.0 × 10^–5^, respectively (Supplementary Table 2). After clumping and harmonization, the number of IVs associated with each psychiatric disorder varies from 3 to 28. For instance, a total of 2,411 IVs are associated with SCZ, and these IVs are categorized into nine bacteria phyla (123 SNPs), 15 classes (208 SNPs), 19 orders (251 SNPs), 30 families (397 SNPs), 102 genera (1,285 SNPs), and 15 species (177 SNPs), respectively. For SCZ, the genus with the largest number of SNPs is *Bifidobacterium* (26 SNPs), followed by *Roseburia* (24 SNPs) and genus with the least number is *Senegalimassilia* (four SNPs). There is no feature containing only one SNP at any level.

The horizontal pleiotropy effect was evaluated at each taxonomic level. For SCZ, only one of 16 IVs for the order *Coriobacteriales* was detected as outlier using the MR-PRESSO outlier test. Similarly, at the family level, two out of 15 IVs in family *Desulfovibrionaceae* and one out of 16 IVs in family *Streptococcaceae* were identified as outliers. After removing pleiotropic SNPs identified by the MR-PRESSO outlier test and the MR-Egger regression, there is no evidence of horizontal pleiotropy of the remaining IVs (both MR-PRESSO Global test *P* > 0.05 and MR-Egger regression *P* > 0.05) (Supplementary Table 3).

### Mendelian Randomization Analysis

Causal association between each pair of bacterial taxon and psychiatric disorder is tested by five MR methods. To take into account multiple-testing correction, the significance threshold for various taxa levels was set to the following: phylum *P* = 5.56 × 10^–3^ (0.05/9), class *P* = 3.33 × 10^–3^ (0.05/15), order *P* = 2.63 × 10^–3^ (0.05/19), family *P* = 1.67 × 10^–3^ (0.05/30), genus *P* = 4.90 × 10^–4^ (0.05/102), and species *P* = 3.33 × 10^–3^ (0.05/15).

**FIGURE 2:**
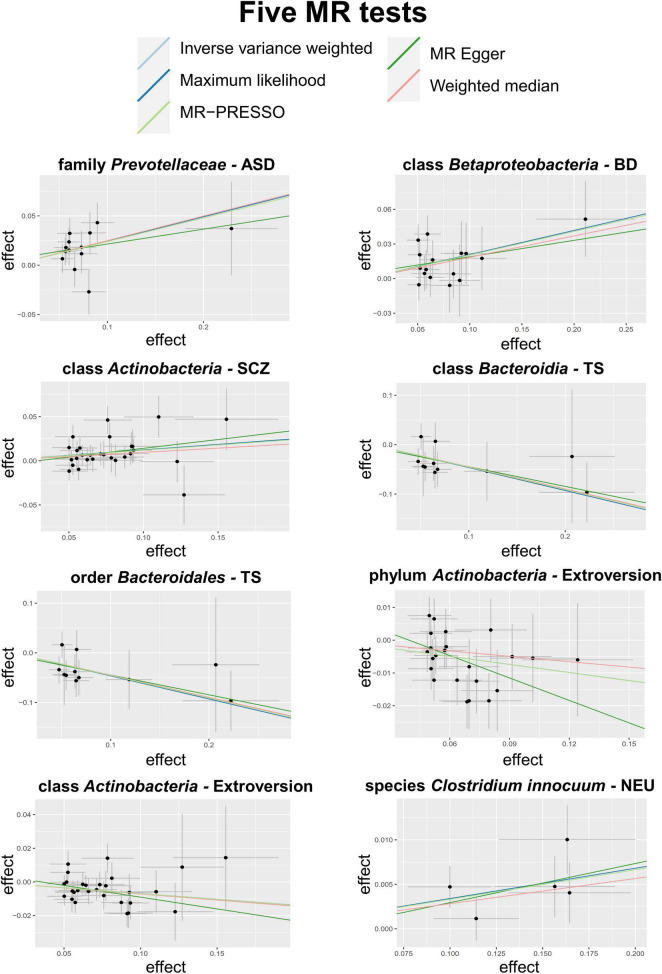
Scatter plots of the 5 MR tests in 8 causal associations from 7 bacterial features to 6 psychiatric disorders/traits. SNP effects were plotted into lines for the inverse-variance weighted test (light blue line), MR-Egger regression (green line), weighted median estimator (red line), MR-PRESSO (light green line) and maximum likelihood estimator (blue line). The slope of the line corresponded to the causal estimation.

A total of eight causal associations from seven bacterial features to six psychiatric disorders/traits were identified by the IVW method (Table 2), including family *Prevotellaceae* with ASD (*P*_*IVW*_ = 5.31 × 10^–4^), class *Betaproteobacteria* with bipolar disorder (BD) (*P*_*IVW*_ = 1.53 × 10^–3^), class *Actinobacteria* with SCZ (*P*_*IVW*_ = 1.33 × 10^–3^), class *Bacteroidia* (*P*_*IVW*_ = 2.51 × 10^–3^), and order *Bacteroidales* (*P*_*IVW*_ = 2.51 × 10^–3^) with Tourette syndrome (TS), phylum *Actinobacteria* (*P*_*IVW*_ = 8.22 × 10^–4^) and class *Actinobacteria* (*P*_*IVW*_ = 1.09 × 10^–3^) with extroversion, and species *Clostridium innocuum* with neuroticism (NEU) (*P*_*IVW*_ = 8.92 × 10^–4^). Scatter plots across various tests are displayed in Figure 2 and Supplementary Figure 1. In total, 234 SNPs are included as IVs of gut microbiota to calculate the causal relationship with psychiatric disorders/traits (Supplementary Table 4). Seven of the eight causal associations are cross-validated by more than two MR tests, demonstrating the robustness of our results. All MR methods produced consistent direction of effect estimates, which strengthens the confidence toward true association (Table 2). Specifically, four bacterial features showed positive causal direction with ASD, BD, SCZ, and NEU, with regression coefficients ranging from 0.03 to 0.24. A total of four bacterial features showed a negative causal direction with TS and extroversion, whose regression coefficients are between −0.46 and −0.07. Of note, genus is a sub-category of family; therefore, the sets of SNPs contained in genus and its relevant family may heavily overlap. For instance, in SCZ, the SNPs of genus *Bacteroides* are within the family *Bacteroidaceae*. Besides, 10 more bacterial features were identified by only one of the five MR tests, as listed in Supplementary Table 5.

**TABLE 2:** Causal estimations of gut microbiota on psychiatric disorders in the MR analysis.

Bacterial taxa (exposure)	Psychiatric disorder/traits (outcome)	No. SNP	R^2^	F	IVW		MLE		MR-Egger		WME		MR-PRESSO	
					*b_xy_*	*P*	*b_xy_*	*P*	*b_xy_*	*P*	*b_xy_*	*P*	*b_xy_*	*P*
Family *Prevotellaceae*	ASD	13	2.61%	37.99	0.24	**5.31 × 10^–4^**	0.25	**6.31 × 10^–4^**	0.15	0.55	0.25	7.81 × 10^–3^	0.24	1.84 × 10^–3^
Class *Betaproteobacteria*	BD	16	3.39%	40.45	0.20	**1.53 × 10^–3^**	0.21	**1.56 × 10^–3^**	0.14	0.44	0.18	0.043	0.20	**1.50 × 10^–3^**
Class *Actinobacteria*	SCZ	28	5.54%	38.60	0.12	**1.33 × 10^–3^**	0.12	**1.12 × 10^–3^**	0.20	0.18	0.10	0.06	0.12	**2.02 × 10^–3^**
Class *Bacteroidia*	TS	11	2.66%	45.82	−0.46	**2.51 × 10^–3^**	−0.46	**3.11 × 10^–3^**	−0.40	0.25	−0.45	0.03	^–^0.46	**1.75 × 10^–3^**
Order *Bacteroidales*		11	2.66%	45.82	−0.46	**2.51 × 10^–3^**	−0.46	3.11 × 10^–3^	−0.40	0.25	−0.45	0.03	^–^0.46	**1.75 × 10^–3^**
Phylum *Actinobacteria*	Extroversion	23	3.36%	27.86	−0.08	**8.22 × 10^–4^**	−0.08	**1.22 × 10^–3^**	−0.23	0.063	−0.05	0.12	^–^0.08	**2.78 × 10^–3^**
Class *Actinobacteria*		28	5.41%	37.68	−0.07	**1.09 × 10^–3^**	−0.07	**1.46 × 10^–3^**	−0.14	0.13	−0.07	0.02	^–^0.07	**1.52 × 10^–3^**
Species *Clostridium innocuum*	NEU	5	1.95%	73.33	0.03	**8.92 × 10^–4^**	0.03	**1.36 × 10^–3^**	0.04	0.43	0.03	0.03	0.03	0.02

*No. SNP is the number of SNPs being used as IVs.*

*R^2^ is the proportion of phenotypic variation explained by used SNPs.*

*F is the value of F statistics to examine the weak instrument bias.*

*b_xy_ is the estimated effect coefficient.*

*s.e. is standard error of estimate coefficient.*

*Significant P-values were marked in bold after multiple-testing correction [phylum P = 5.56 × 10^–3^ (0.05/9), class P = 3.33 × 10^–3^ (0.05/15), order P = 2.63 × 10^–3^ (0.05/19), family P = 1.67 × 10^–3^ (0.05/30), genus P = 4.90 × 10^–4^ (0.05/102) and species P = 3.33 × 10^–3^ (0.05/15)].*

*IVW, the inverse-variance weighted test; MLE, the maximum likelihood estimator; WME, the weighted median estimator; ADHD, attention deficit/hyperactivity disorder; ASD, autism spectrum disorder; BD, bipolar disorder; SCZ, schizophrenia; OCD, obsessive-compulsive disorder; TS, Tourette syndrome; NEU, neuroticism; SA, suicide attempt.*

Instrumental variables for each identified features can explain 1.95–5.54% of the variance in each feature and 0.003–0.53% of the variance in the corresponding psychiatric disorders/traits, respectively. The F statistics for all IVs are larger than 10, indicating no evidence of weak instrument bias. Furthermore, the MR Steiger directionality test revealed that the variances explained by included SNPs of bacterial exposure are larger than psychiatric outcome, implying the true causal associations directionally (Supplementary Table 3). Q statistics of the IVW test and the MR-Egger regression showed no evidence of heterogeneity at the identified results (Supplementary Table 3). Forest plots of causal effects using single SNP showed that none of them is extremely significant for association with psychiatric disorders/traits (Supplementary Figures 2A, 3A), and the leave-one-out sensitivity analysis demonstrated no single SNP driving the causal association signal (Supplementary Figures 2B, 3B).

The results of the reverse MR analysis, as listed in the Supplementary Table 6, showed no evidence of causal effect from psychiatric disorders/traits to identified bacterial features after multiple-testing correction (*P* < 0.05/18 = 2.78 × 10^–3^).

Some factors, such as chronic bowel diseases, may affect the association between gut microbiota and psychiatric disorders. To check the potential influence of confounding factors, we identified several sub-types of chronic bowel disease, including irritable bowel syndrome, inflammatory bowel disease, ulcerative colitis, Crohn’s disease, bowel problem, other non-infective gastroenteritis and colitis, and other functional intestinal disorders categories. We retrieved the associations of the identified IVs with each type in the United Kingdom Biobank summary statistics through the GeneATLAS website^4^. After multiple-testing correction, the results showed that none of the associations is significant, indicating limited confounding effect of chronic bowel diseases, as listed in the Supplementary Table 7. We further excluded IVs associated with any one of the chronic bowel diseases at a nominal level (*P* < 0.05) and re-perform the MR analysis using the remaining IVs. The results remain significant at the eight identified causal associations. Meanwhile, neither the MR-PRESSO test nor the MR-Egger regression test showed evidence of horizontal pleiotropy (both *P* > 0.05). Together, these results implied that the identified causal associations were unlikely to be mediated by chronic bowel disease.

## Discussion

In the current study, we conducted MR analyses to evaluate the potential causality between the gut microbiota and 15 psychiatric disorders/traits. Using large-scale summary statistics from microbiome GWAS and 15 psychiatric disorders/traits GWAS, we identified seven bacterial features that were causally associated with six psychiatric disorders/traits.

The positive association between family *Prevotellaceae* and ASD is in line with previous findings (Qiao et al., 2018; Dan et al., 2020). *Prevotellaceae* is characterized as a propionate-producing bacterium. Previous studies have found that propionate, as an enteric metabolite produced by gut microbiota, induced social abnormalities, cognitive impairments, sensorimotor dysfunction, and exacerbated ASD symptoms after intracerebroventricular injection (Shultz et al., 2015). Other studies have also manifested the consistent inference that changes in brain tissue after propionate administration result in conditions similar to ASD patients, such as reactive astrogliosis and oxidative stress (Thomas et al., 2012).

In accordance with the previous studies, class *Actinobacteria*, as a gram-positive bacterium, showed a positive causal association with SCZ, and class *Betaproteobacteria* has a positive direction on BD. For instance, patients with SCZ and other psychotic disorders have a higher abundance of *Actinobacteria* (Zheng et al., 2016; Li et al., 2020; Vindegaard et al., 2020), which is also supported by animal models (Dunphy-Doherty et al., 2018). Members of *Betaproteobacteria* were found to be more abundant in a mouse model of psychiatric diseases, whereas they were strongly correlated with increased gut permeability and intestinal chronic inflammation in humans, which may affect mental health or brain development *via* the MGB axis (Bauerl et al., 2018; Chen et al., 2018).

Consistent with previous literature, our result showed that class *Bacteroidia* and its child taxon, order *Bacteroidales*, have a negative effect on TS. Both *Bacteroidia* and *Bacteroidales* are correlated with the tryptophan hydroxylase-II (TPH2) serotonin pathway (Liu et al., 2020). In animal experiments, deficits and excess of TPH2 activity may induce significant behavioral disturbances and catalepsy, whereas the human *TPH2* gene is related to psychiatric disorders (Kulikova and Kulikov, 2019). Moreover, serotonin, one of the main brain neurotransmitters, plays an important role in promoting immunity and reducing inflammation in mucosal infections (Gao J. et al., 2018). Thus, decreased serotonin concentrations in the brain and cerebrospinal fluid in Tourette patients might be implicated in the pathogenesis of TS (Mossner et al., 2007). In a FMT study, the abundance of *Bacteroides coprocola* was reduced in TS patients, but its restoration could improve tic symptoms (Zhao et al., 2020). Personality traits, such as extroversion and NEU, affect fundamental behavior patterns and have been related to mental disorders. In the present study, phylum *Actinobacteria* and its child taxon, order *Actinobacteria*, both have a negative effect direction on extroversion, whereas species *Clostridium innocuum* has a positive effect on NEU.

In addition to the above causal associations identified by IVW test, several intriguing results were identified by other MR tests, including families *Christensenellaceae* and *Methanobacteriaceae*, both of which have negative effects on OCD and SA. These associations are broadly supported by previous studies. For instance, it is accepted that SCFA butyrate might suppress the inflammation and oxidative damages in colon and brain, alleviating cognitive impairments, behavioral disorders, and gastrointestinal disorders (Peruzzotti-Jametti and Pluchino, 2018). Family *Christensenellaceae*, as a gram-negative, strictly anaerobic, and SCFA-producing taxon (Waters and Ley, 2019), could increase the concentration of butyrate in colon, potentially alleviating colitis-related OCD behaviors by diet in humans (Nagpal et al., 2019). It was also found to be positively correlated with cognitive ability in mice (Gao L. et al., 2018). Whereas *Methanobacteriaceae*, a dominant methanogenic archaeon, can increase levels of SCFAs in the colon (Samuel and Gordon, 2006) and likely has beneficial psychological effects *via* SCFAs such as migraine reduction in elderly women, which could partly explain the protective effect of this taxon against suicide (Chen et al., 2019).

There are many population-based observational studies and their meta-analyses for the association of gut microbiota with psychiatric disorders (Strati et al., 2017; Ma et al., 2019; Valles-Colomer et al., 2019; Hua et al., 2020; Iglesias-Vazquez et al., 2020; Nikolova et al., 2021). Among them, Nikolova et al. (2021) meta-analyzed 34 case-control studies in a total of 1,519 psychiatric patients versus 1,429 normal controls and found no difference in the diversity of gut microbiota. The meta-analysis attempts to resolve a controversial scientific question such as if an association between two conditions exists, whereas the causal nature of such association is unknown. Fundamentally different from meta-analysis, MR analysis, on the other hand, is an approach statistically inferring the causal nature of an association observed in a cross-sectional study.

This study has advantages in several aspects. First, the identified causal relationship may provide candidate bacteria for subsequent functional studies. Second, we comprehensively studied up to 15 common psychiatric disorders. In a previous study, Zhuang et al. (2020) studied three psychiatric disorders and revealed that order *Enterobacteriales* and family *Enterobacteriaceae* were causally associated with a higher risk of schizophrenia, and increased class *Bacilli* was causally associated with a higher risk of major depressive disorder.

There are also certain limitations in this study. First, gut microbiota GWAS is still in its infancy in terms of sample size; therefore, the number of associated loci is relatively small compared with that for psychiatric disorders. Second, because of the small sample size and insufficient power for microbiome GWAS, there may not be enough IVs for certain bacterial features at genus or species level. As a compromise, we analyzed features at a higher level (phylum, class, order, or family). When microbiome GWAS will eventually be equipped with sufficient sample size, these more specific features will hopefully be identified at a finer resolution (Thomas, 2019). Third, to maximize sample size and statistical power, GWAS of gut microbiota and psychiatric disorders/traits analyzed in this study might originate from multi-ancestry samples. Thus, the results should be interpreted with caution.

In conclusion, we comprehensively assessed the potential causal association between gut microbiota and a series of psychiatric disorders/traits. Four bacterial features showed positive causal direction with ASD, BD, SCZ, and NEU, whereas another four bacterial features showed a negative causal direction with TS and extroversion. This study may be useful in providing new insights into the development mechanism of microbiota-mediated psychiatric disorders.

## Data Availability Statement

The original contributions presented in the study are included in the article/Supplementary Material, further inquiries can be directed to the corresponding author/s.

## Ethics Statement

The studies involving human participants were reviewed and approved by all studies were approved by respective institutional review boards (IRBs). No new IRB approval was required. Written informed consent to participate in this study was provided by the participants’ legal guardian/next of kin.

## Author Contributions

LZ and Y-FP designed the study. LZ, QX, and J-JN collected the data. J-JN and QX analyzed the data. S-SY, B-XH, HZ, X-TW, G-JF, Y-FP, MZ, QX, and J-JN performed the literature search. J-JN drafted the early version of the manuscript. LZ and Y-FP jointly supervised the study. All authors were involved in writing the manuscript and had final approval of the submitted and published versions.

## Conflict of Interest

The authors declare that the research was conducted in the absence of any commercial or financial relationships that could be construed as a potential conflict of interest.

## Publisher’s Note

All claims expressed in this article are solely those of the authors and do not necessarily represent those of their affiliated organizations, or those of the publisher, the editors and the reviewers. Any product that may be evaluated in this article, or claim that may be made by its manufacturer, is not guaranteed or endorsed by the publisher.

## Footnotes

1
http://www.mibiogen.org

2
https://github.com/MRCIEU/TwoSampleMR

3
https://github.com/rondolab/MR-PRESSO

4
http://geneatlas.roslin.ed.ac.uk/phewas

## Abbreviations

ADHD: attention deficit/hyperactivity disorder
ASD: autism spectrum disorder
SCZ: schizophrenia
FMT: fecal microbiota transplantation
MGB: microbiota–gut–brain
RCT: randomized controlled trial
MR: Mendelian randomization
IV: instrumental variable
SNP: nucleotide polymorphism
SCFA: short-chain fatty acid
GWAS: genome-wide association study
IRB: institutional review board
LD: linkage disequilibrium
IVW: inverse-variance weighted
MLE: maximum likelihood estimator
WME: weighted median estimator
BD: bipolar disorder
TS: Tourette syndrome
NEU: neuroticism
OCD: obsessive-compulsive disorder
SA: suicide attempt
TPH2: tryptophan hydroxylase-II.
