# Supplementary material for: Gut Microbiota and Psychiatric Disorders: A Two-Sample Mendelian Randomization Study
Source: Front Microbiol. 2022 Feb 4;12:737197. doi: 10.3389/fmicb.2021.737197 (PMC8856606; doi:10.3389/fmicb.2021.737197)

**Figure legends**

**Supplemental Figure 1. Scatter plots of the 5 MR tests in 10 causal associations from bacterial features to 6 psychiatric disorders/traitsidentifiedinothercomplemental MR methods.**

SNP effects were plotted into lines for the inverse-variance weighted test (lightblueline), MR-Egger regression (green line), weighted median estimator (red line), MR-PRESSO (lightgreen line) andmaximum likelihood estimator (blue line). The slope of the line corresponded to the causal estimation.

**Supplemental Figure 2. Forest plot of causal effects (A) and MR leave-one-out sensitivity analysis (B) for 8 causal associations from 7 bacterial features to 6 psychiatric disorders/traitsidentified in the IVW test.**

(A) The causal effect of gut microbiota on psychiatric disorders/traits was estimated using each SNP singly using the Wald ratio and using all SNPs using the MR Egger and IVW methods. (B) Leave-one-out sensitivity analysis represents the MR analysis excluding the particular SNP usingthe IVW test.

**Supplemental Figure 3. Forest plot of causal effects (A) and MR leave-one-out sensitivity analysis (B) for10 causal associations from bacterial features to 6 psychiatric disorders/traitsidentifiedin other complemental MR methods.**

(A) The causal effect of gut microbiota on psychiatric disorders/traits was estimated using each SNP singly using the Wald ratio and using all SNPs using the MR Egger and IVW methods. (B) Leave-one-out sensitivity analysis represents the MR analysis excluding the particular SNP usingthe IVW test.

**Supplemental Figure 1**

**
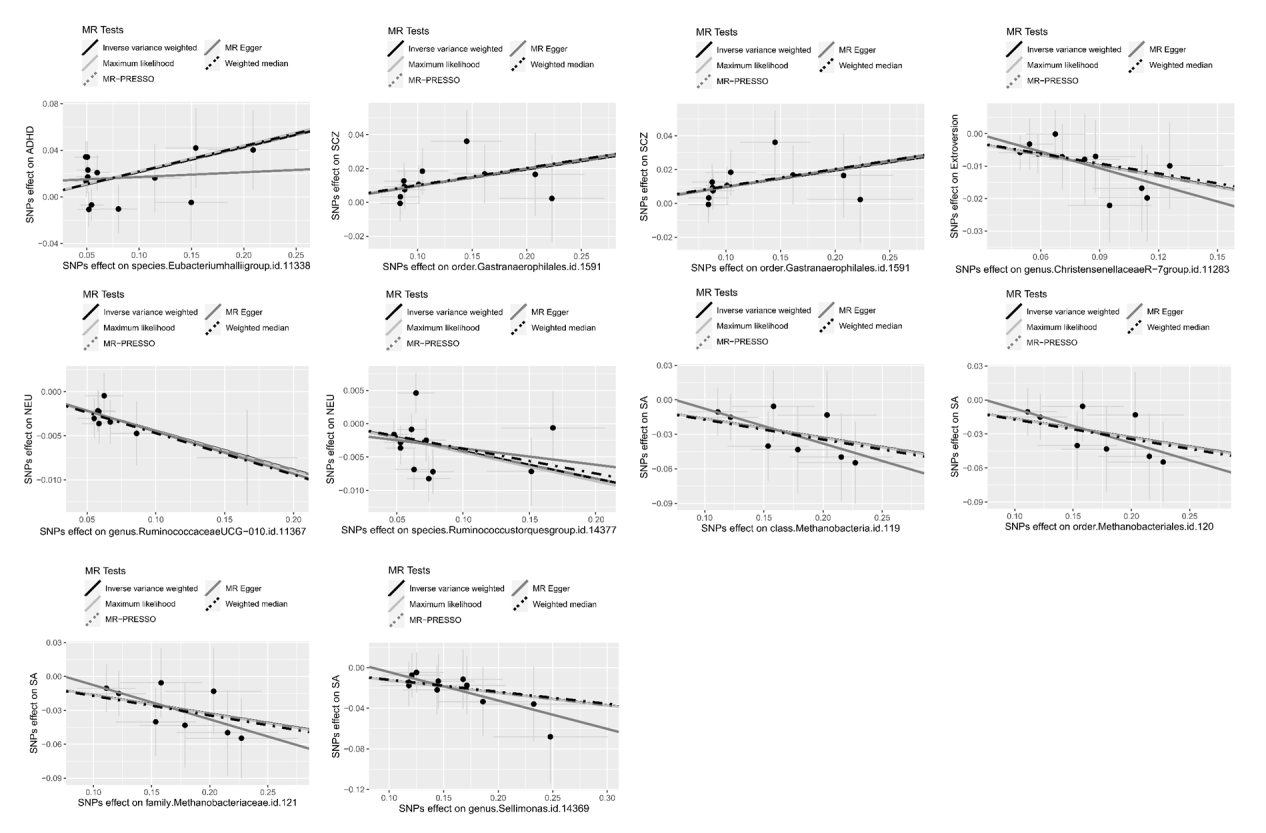
**

**Supplemental Figure 2**

**
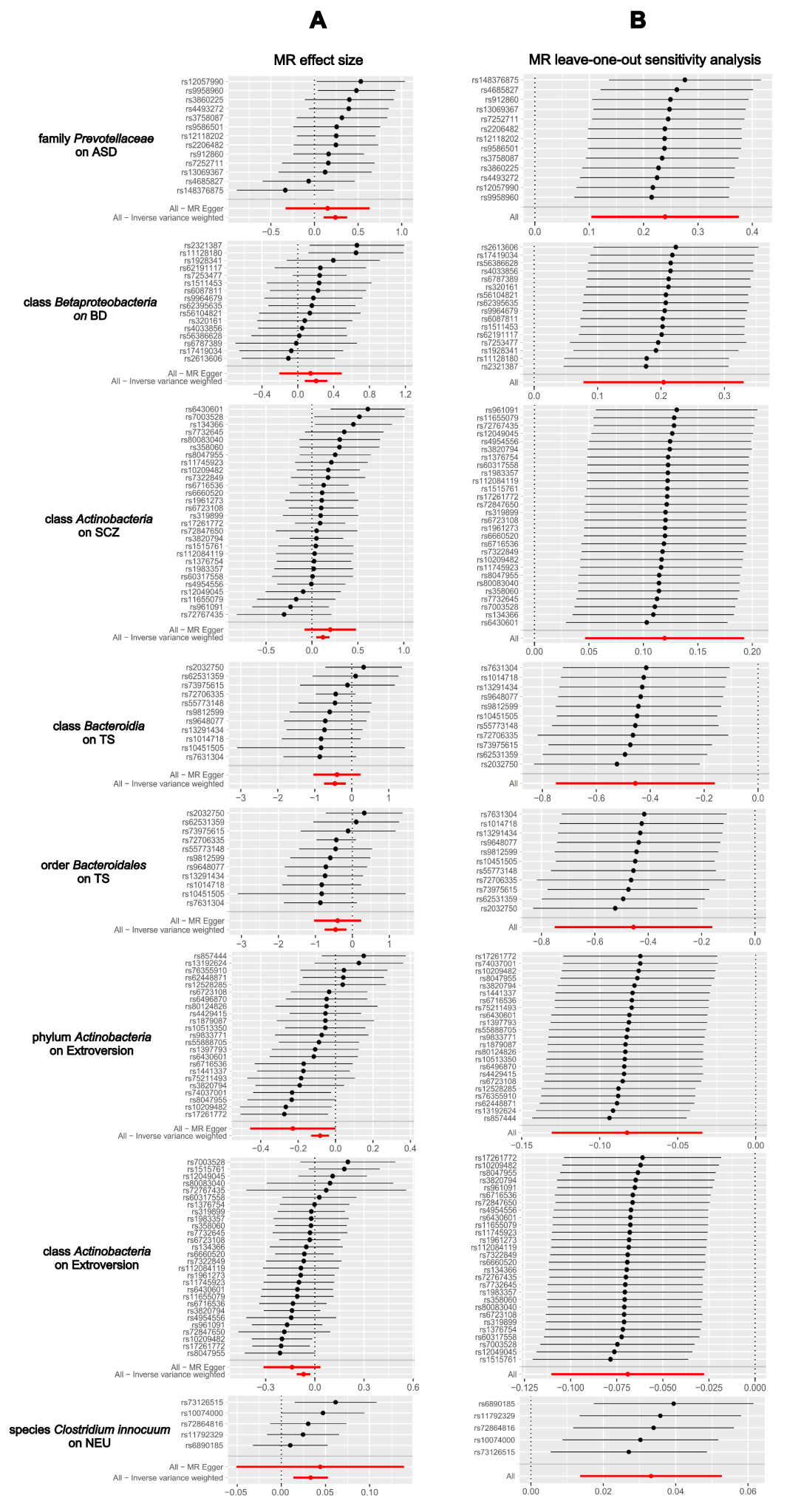
**

**Supplemental Figure 3**


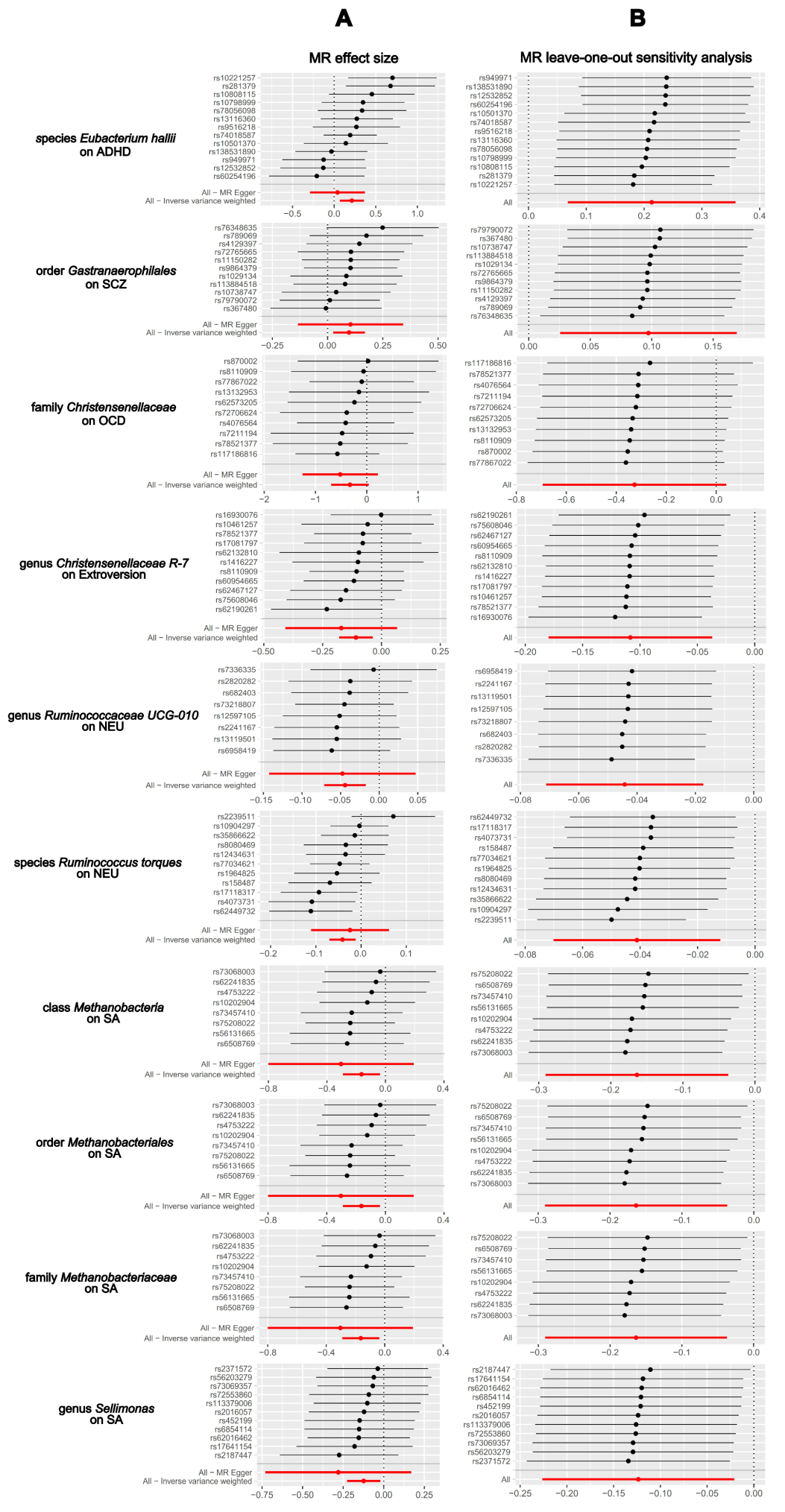

Supplement: Supplementary file 2 [file Data_Sheet_2.docx]
